# Voices of formerly enslaved: A new text corpus of narratives by formerly enslaved persons

**DOI:** 10.1038/s41597-026-07340-x

**Published:** 2026-04-30

**Authors:** Irene Elmerot, Leif-Jöran Olsson, Klas Rönnbäck

**Affiliations:** 1 Department of Swedish, Multilingualism, Language Technology, University of Gothenburg, Box 100, SE-405 30 Gothenburg, Sweden; 2 Department of Economy and Society, University of Gothenburg, Box 100, SE-405 30 Gothenburg, Sweden

**Keywords:** Interdisciplinary studies, Social sciences, History

## Abstract

These data consist of newly OCRed and annotated narratives, both autobiographical texts written by, and interviews with, formerly enslaved persons of African descent in the United States of America and the Caribbean, including extensive time-related and geographical metadata. The texts authored by these individuals span from the years 1795 to approximately 1900, while the interviews were conducted in the 1930s. The former are written in standardised English from that time, whereas the latter often are written down in mediated, vernacular form, causing issues in lemmatisation and part-of-speech tagging. The aim is to create openly accessible corpora that can be utilised for the purpose of researching how these formerly enslaved persons described their own lives.

## Background & Summary

The aim of this research project is to study the living conditions of enslaved and/or recently emancipated persons who resided in the United States of America (and, to a small extent, in the Caribbean). This research will be performed using an annotated corpus of texts that consists of different parts, or subcorpora. The data presented in this article was derived from two of these text parts: autobiographies by and interviews with such persons.

The current state-of-the-art research on historical living standards relies on quantitative methods for analysing large samples of historical populations^1^. This approach has generally overlooked the testimonies of enslaved individuals, despite their significant value. These first-hand accounts offer crucial insights into the social history of slavery, detailing the experiences of enslaved people both during and after their enslavement. While some historians have qualitatively analysed evidence of these experiences^2,3^, a systematic analysis of a large dataset documenting the lived experiences of formerly enslaved individuals has not yet been conducted. These testimonies also matter linguistically, since they can shed light on the language used by this social strata. Previous language studies, such as Schneider^4^, have used only a few of these data. Future research, however, could leverage the full dataset to yield more robust findings.

Due to common interests, a collaboration with the *Baquaqua: The Afro-Diasporic Text Corpus Project* (https://library.morgan.edu/aatc) has been initialised, and discussions on best tool practice have been performed both online and on site at Morgan State University Library. For our joint purposes, one subcorpus, AATC, in our dataset^5^ consists of public domain texts they are also working on. All the corpora (the whole and its parts), made available for open access, have the additional advantage that they can be put to use for much other research, studying a variety of research questions, in both the Humanities and the Social Sciences.

The sources of data used for this research are interviews with and autobiographies by formerly enslaved persons, in the previous research often called “slave narratives” more generally. In such records, the formerly enslaved individuals describe how they remember their own lived experience of slavery. Such records can be found in various historical archives or in published form, from various countries and in various languages^6,7^. All sources used for this article and our corpus version 0.1^5^ are openly accessible.

Two key sets of sources were used for the pilot corpus, as seen in Table 1. The first set of records comprised autobiographies published by individuals who had previously experienced enslavement. These autobiographies had previously been collated and digitised in a separate project, entitled Documenting the American South (here abbreviated DocSouth), by the University Library at the University of North Carolina at Chapel Hill (https://docsouth.unc.edu/index.html). It is estimated that more than 200 such autobiographies were published during or in the aftermath of the period when slavery was legal. In this study, 188 out of 446 texts from the DocSouth collection have been included; all the first-person narratives by African-Americans in this collection. These texts are from various states within the USA and from some parts of the Caribbean. The metadata currently relies on the geographical metadata from the DocSouth project. Exact details of the current known geography is found in the Metadata file, in the different variables named Geography.

**Table 1:** Source material size and status for corpus version 0.1.

Sources	Estimated sample size of collected texts	Status	No. of tokens	Metadata variables
DocSouth slave autobiographies etc.	188 books	Wholly digitized (including optical character recognition, OCR).	7.2 million	Years, Genre, Geography, Main person, Gender, Pages, Other pages, Standard variety pages, Spoken variety pages, Utterance pages.
FWP ex-slave interviews	2243 interviews	Wholly digitized (including OCR).	3.0 million	Years, Genre, Geography, Main person, Gender, Pages, Other pages, Standard variety pages, Spoken variety pages, Utterance pages, Interviewer, Editor

The metadata variables are examples of the existing metadata.

The second set of records was interviews conducted by the Federal Writers’ Project (FWP)^8^. These interviews were conducted in the 1930s with individuals who had been enslaved during their childhood or early adulthood. It is estimated that the total number of interviews amounts to several thousands^4^. In future releases, therefore, the intention is to add more interviews. The metadata are described in more detail in the Methods section. For the pilot study and corpus version 0.1^5^ described in this article, 33 volumes collected in 17 states (as seen in Table 2) were included, with a total of 2243 interviews.

**Table 2:** The publishing US state and number of interviews per FWP volume included in corpus 0.1.

Volume	State	Interviews
Vol01	Alabama	**128**
Vol02	Arkansas	**740**
- Vol02_01		102
- Vol02_02		103
- Vol02_03		112
- Vol02_04		105
- Vol02_05		120
- Vol02_06		117
- Vol02_07		81
Vol03	Florida	**51**
Vol04	Georgia	**167**
- Vol04_01		42
- Vol04_02		51
- Vol04_03		52
- Vol04_04		22
Vol05	Indiana	**6**
Vol06	Kansas	**3**
Vol07	Kentucky	**8**
Vol08	Maryland	**22**
Vol09	Mississippi	**26**
Vol10	Missouri	**89**
Vol11	North Carolina	**173**
- Vol11_01		88
- Vol11_02		85
Vol12	Ohio	**34**
Vol13	Oklahoma	**74**
Vol14	South Carolina	**335**
- Vol14_01		89
- Vol14_02		85
- Vol14_03		82
- Vol14_04		79
Vol15	Tennessee	**26**
Vol16	Texas	**290**
- Vol16_01		76
- Vol 16_02		72
- Vol16_03		74
- Vol 16_04		68
Vol17	Virginia	**14**
**Total**		**2243**

As seen in Table 2, the Arkansas interview volumes comprise a large part, 7 of the total, with Georgia, South Carolina and Texas being equally represented by 4 volumes. Different volumes include different numbers of interviews. Volume 7, compiled in Kentucky, is a composite book that incorporates interview transcripts alongside extraneous material. For the present release (v0.1) we extract and present the individual interviews from that volume; the so-called combined interviews will be included in v1.0. Material that is not interview-based is excluded from the project.

The present metadata file, for the FWP volumes in version 0.1 of the corpus, only includes the US state and the names of the interviewees, with one, representantive, volume. For this one volume (Vol04_03 in Table 2), we manually added a more specific place, recorded by the interviewer or editor on the first page of an interview, as well as details on the language variety on each page. In later versions of the corpus, however, there will be such metadata for all volumes, and geographical metadata for both datasets will be improved by adding places mentioned in the texts after an Named Entity Recognition analysis.

Due to the age of these autobiographies, copyright protection for the texts has expired. As for the FWP records, output by federal employees in the USA is not covered by copyright. The original records collected for the corpus are thus all in the public domain, available for research purposes. The copyright holders of the digitised versions of these DocSouth records have granted us permission to include them in the corpus for research purposes. The records from the FWP had likewise been digitised, by the U.S. Library of Congress, and made available online for research in their “Born in Slavery” collection of documents (https://www.loc.gov/collections/slave-narratives-from-the-federal-writers-project-1936-to-1938/about-this-collection).

## Methods

The creation of the corpus and its resources is a progressive process. In certain cases, steps are taken to test a specific workflow, and these tests may be removed if the results are deemed unsatisfactory or unuseful. To ensure the robustness of this process, it has been configured to operate on an iterative basis, with each step having its own version. The process thus entails the progressive enhancement of the project’s corpus resources, using additional components and workflows as required, thereby ensuring the robustness of the progress. The different editions will undergo versioning during the process, to facilitate the experience for users. This exercise was first initiated using the modest AATC corpus and a part of our own data, with the objective of surveying the potential workflows and existing tools to create our corpus. This approach was informed by our prior experiences with open standards and XML tooling.

The processing of all corpus data, inclusive of the automatically extractable metadata, was conducted utilising the *Sparv* 5.3.1^9^ Pipeline. Manually extractable metadata will be included in the process later during the project. All extractions, including the OCRing to obtain TEI-XML for further processing, were facilitated by *Tesseract* (v5.4.1, https://github.com/tesseract-ocr) as a *Sparv* plugin in the pipeline. Furthermore, comparisons were conducted to a) reveal whether our automated analyses were improved or not, and b) reveal whether or not a specific step in the process worked better than others. These comparisons were partly conducted to identify improvements within individual analyses, and partly to find possible trends in which analysis works better for our different text types.

One annotator proceeded to manually proofread the first iteration, incorporating variables such as a corrected part-of-speech (PoS) and a correct lemma. The annotator also provided commentary on instances where the PoS could be considered uncertain or where a single token might possess multiple lemmata. An example is the token “de”, which may have either the lemma “the” or the lemma “they”. A more prominent example is the lemma “master”, a typical keyword for these texts, that was found to have about 20 different tokens in the first round, including the standardised spelling but with capital letters only or plural form. These corrections were then fed back to train a new annotation model. The pilot project annotations are available in the “Annotations” folder, in different formats such as csv, conllu and xml (10.23695/P5HW-DR52).

The original texts (pdfs) were subjected to a manual examination to identify and categorise pages based on their linguistic characteristics. Metadata curation, encompassing both descriptive and content-related data, constituted a significant phase of the research methodology. The implementation of unique identifiers streamlined subsequent processes, including data validation, entry, extraction, and testing. A distinction was made between pages such as colophons (included in the variable name Other_pages), pages employing a standardised linguistic register (included in the variable Standard_variety_pages), and those featuring transcribed spoken language. The latter exhibited orthographic variations not present in standard English dictionaries, exemplified by spellings such as “mout” for “might” and “gwine” for “going.” (Although Schneider^4^ considers the term ‘dialect’ to be a neutral term, we consider the term ‘variety’ to describe these discrepancies better). Another illustration of this phenomenon can be observed in phrases such as “Dey bilt dem a house” for “They built themselves a house”. In most cases, this metadata variable, called “Spoken_variety_pages”, refers to pages with a transcribed spoken variety, where vernacular speech is cited by the interviewer or editor of text. It is important to note that this speech is mediated by the interviewer/editor, and that this process of mediation sometimes entailed substantial editing of the transcribed speech^10^. A third spelling-related variable pertains to instances where, in a volume with otherwise mainly standardised variety English, a part of a page is composed of speech or dialogue, transcribed to emulate the African-American vernacular English of the era. This metadata variable is called Utterance_pages.

Subsequently, the *Sparv* pipeline employs the lemma annotations to generate input for e.g. the word embeddings model generation. The Named Entity Recognition (NER) annotations are utilised for subsequent steps as input.

**Fig. 1:**
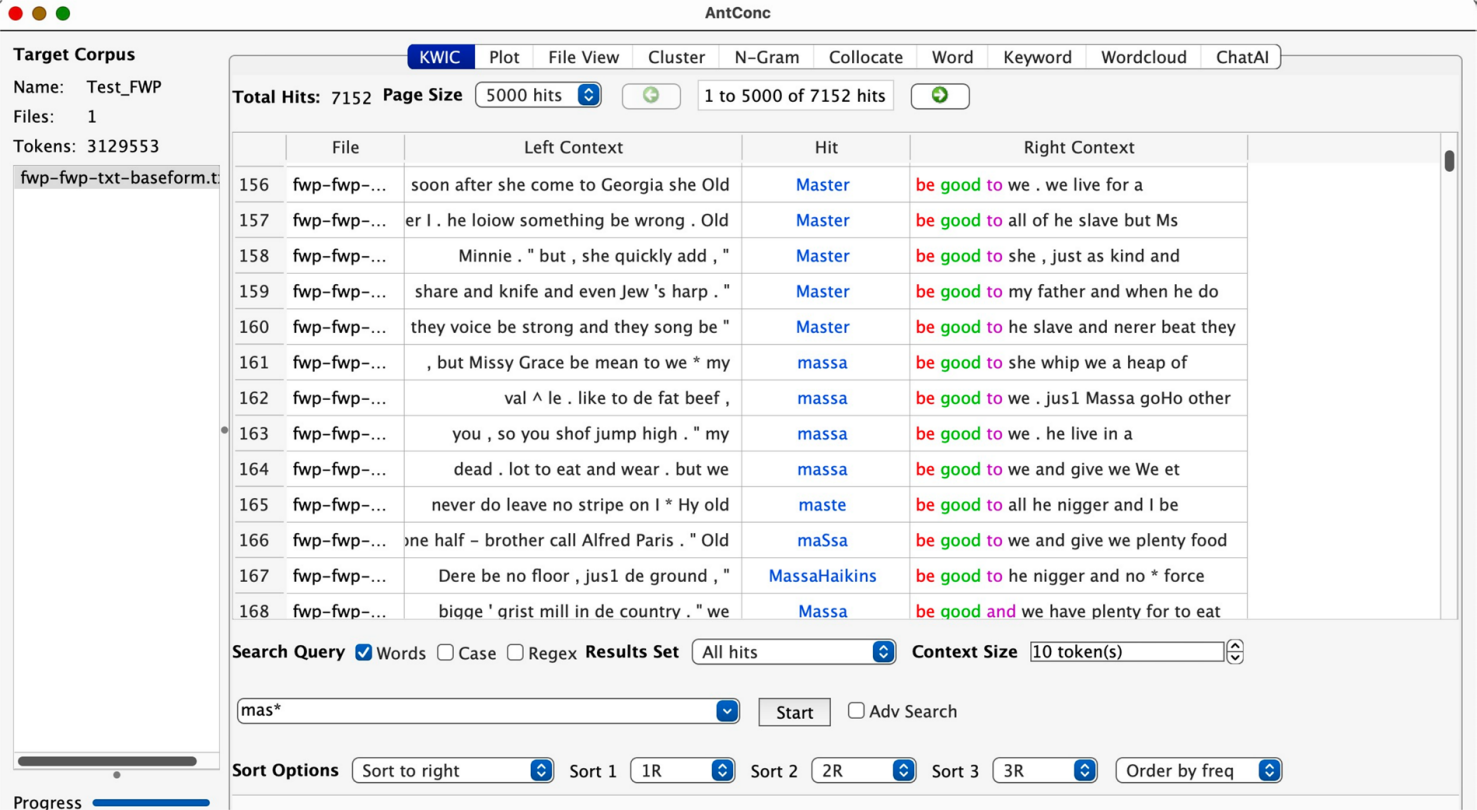
The first OCR version of FWP interviews as seen in AntConc.

The steps explained above are taken not only to create a corpus that includes as many of these narratives as possible, but also because they are necessary to create a corpus that can be used through various corpus apps. Initially, the outputs are primarily created for *Corpus Workbench*-based tools, for example CQPweb, but there are also csv files that may be used in other tools. An example is shown in Fig. 1, where an early version of the FWP subcorpus, the transcribed interviews, has been opened in AntConc^11^. This concordance example shows not only that there are different spellings of one and the same word (“master”), but also that the other words have plenty of issues, such as the previous OCR having converted letters to numbers.

In Fig. 1, we see a search for mas*, meaning all tokens that start with the letter combination mas, to include more spellings of the word “master”. In addition, throughout all of these concordance lines, the token “be” should be interpreted as different forms of the verb “to be”, most often “is”, but it could likely also be “was”, “used to be” or even “has been”. These two words illustrate two of the data issues – lemmatisation and misinterpreted OCR – that we aim to solve with this project, one step at the time, before we start analysing the texts themselves. Files to be used in stand-alone software such as this are found in the “Sources” folder. The folders there contain source material used to perform the analyses found in the “Analyses” folder. Instructions are available, and will be updated during the rest of the project, in the “Documentation” folder in our repository (10.23695/P5HW-DR52).

For future releases of the corpus, we are adding text type classifier models, but also topic modelling data, semantic and frame data, and sentiment annotations. We expect to have SQL-tables and other structured data for use within different tools before 2027. There will be information on syntactical relations to use in for example *Korp*’s “word pictures” (word sketches), but also resources in other formats than the current, for exploration in other *Corpus Workbench*-based tools.

## Data Records

The dataset^6^ and related resources are available at a Språkbanken Text repository (https://spraakbanken.gu.se/en/resources/votfe-pilot) with this section being the primary source of information on the availability and content of the data being described. The data belonging to corpus version 0.1 have the following structure:

- Analyses material:
  - Word2vec and fasttext models.
- Annotations:
  - Mostly token and sentence level, inlined for all parts of the entire corpus:
    - NER entities.
    - Lemmata (called baseforms in some formats), Parts-of-Speech.
    - Syntactic dependencies.
  - A frequency list for each subset of the corpus.
  - Resources in vrt format for exploration in Corpus Workbench-based tools.
- Stand-alone output:
  - The whole, fully annotated corpus in different file formats (xml etc.).
  - The fully annotated sub-corpus files.
  - The whole corpus with less annotations in relevant formats (txt, conllu, csv etc.).
  - The less annotated sub-corpus files.
- Documentation:
  - Videos, for example on how to open a (sub)corpus file in different corpus tools.
  - Written instructions
    - how to reproduce (within our or other corpus/NLP tools),
    - how to extract for partitioning on different variables or annotations.
- Illustrations, figures and pictures that we have used or will use in articles and presentations.
- Metadata, semi-automatically extracted and manually curated, as well as manually produced. The metadata file includes a codebook data sheet, where all variables are explained.
- License, CC-BY-SA 4.0.

## Data Overview

**Fig. 2:**
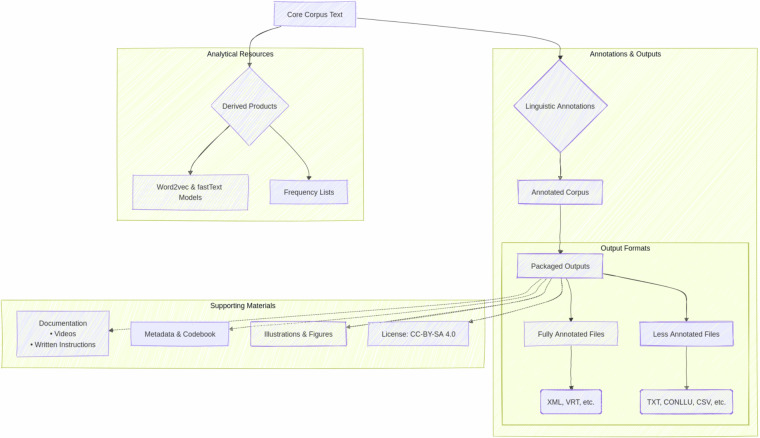
Data flowchart overview.

The dataset and codes on the project’s repository^5^ is version 0.1. An overview of the data is found in Fig. 2.

## Technical Validation

The digitised source texts for the corpus have been restructured at different times. The different pdf and xml files stored at the DocSouth and FWP websites have previously been restructured by their respective projects. However, it was found that the digitisation contained a substantial number of errors. With advancements in technology since the initial digitisation, state-of-the-art optical character recognition (OCR) will now be employed to create a more accurate and consistent corpus. We have conducted new OCR tests on these records, and will use the advantages of the different test versions to create a more robust and consistent final corpus. Manual annotation has been employed to train classifiers, with inter-annotator agreement measured using Fleiss’ ϰ. This manual annotation has especially improved the lemmatisation process. The statistical significance of samples is determined through manual evaluation of automatically annotated parts. A comparison of different versions is then performed to identify any additions, removals, or omissions. This pilot project has revealed a substantial number of inconsistent normalisations. This pilot project has identified a substantial number of inconsistent normalisations.

**Fig. 3:**
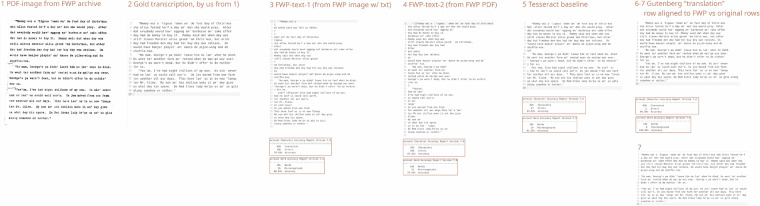
Comparison images showing different sources of text and a gold transcription. 1) A pdf page from the FWP project’s website. Paragraphs are here marked by handwritten lines. 2) Gold transcription, manually corrected, of the text from 1). 3) FWP text variant 1, taken from the FWP website tab “Images with text”. 4) FWP text variant 2 as embedded in the website’s pdf version. 5) Tesseract Baseline OCR using its default settings (Tesseract v5.4.1). 6) Gutenberg “translation” where the rows were aligned by us to match the other images in this figure. 7) Gutenberg translation with original row numbering (note the difference from the image and other sources’ row numbering).

The text excerpt in Fig. 3 is from volume 4, book 3 of the Federal Writers’ Project collection (https://www.loc.gov/resource/mesn.043/?sp = 117&st = text&r = -0.301,-0.08,1.603,1.603,0 and digital id https://hdl.loc.gov/loc.mss/mesn.043).

In images 3), 4), 5), and 6) of Fig. 3 the Character Error Rate (CER) and Word Error Rate (WER) scores are overlaid on the text images. The term “character error” means that two single characters differ, and “word error” that two tokens differ between two images.

The Tesseract baseline image 5), with no adaptation (using the default settings) gave a CER of 94.92 percent and a WER of 91.35 percent. Compared to the versions from FWP, images 3) and 4), this tells us that such a modern OCR tool is useful for most cases, even without modification. The OCR from FWP in images 3) and 4) also contains chunks of text in the wrong order, as well as text from other paragraphs. An example of this from image 3) are lines 4, 5 and 6. This is less obvious in image 4), but lines 3, 4 and 5 are jumbled. As these texts extracted from the PDFs exhibit similar behaviour but at the same time contain different errors, we can probably expect further error variants within other text samples, perhaps because they are produced with different engines and at different times. We have therefore prepared to improve the OCR using the Tesseract plugin.

When we compared this initial outcome with the Gutenberg curated versions added later, images 6) and 7), we can see that the WER is 100 percent. However, their versions have been proofread, so that the word accuracy is 100 percent is somewhat expected. Unfortunately, the Gutenberg versions are also inconsistently normalised. In these three paragraphs, the text happens to be identical, whereas in other samples there may be different corrections and even comments. This, on the other hand, gives us the opportunity to treat the FWP archive versions, which are also available from Gutenberg, as “translations” of each other, or comparable texts, for the whole sub-corpus. We expect the available Gutenberg versions to be much fewer for the future project leading to corpus version 1.0, since they have not included all volumes and pages in their collection. However, we have only used them as reference, to note the difference between our OCR and theirs.

The evaluation of metadata variables is conducted through the utilisation of consistency tests, or positive and negative tests, for variables such as the interviewee’s or author’s year of birth. These tests are used to solve problems such as whether the data can be extracted automatically. A negative test can determine whether any values are empty when they should not be. The equivalent positive test confirms that the value is not only present, but also the same as in the source file. While birth years may be uncertain, the concept of (un)certainty is not yet a factor in the pilot study’s metadata assessment.

**Fig. 4:**
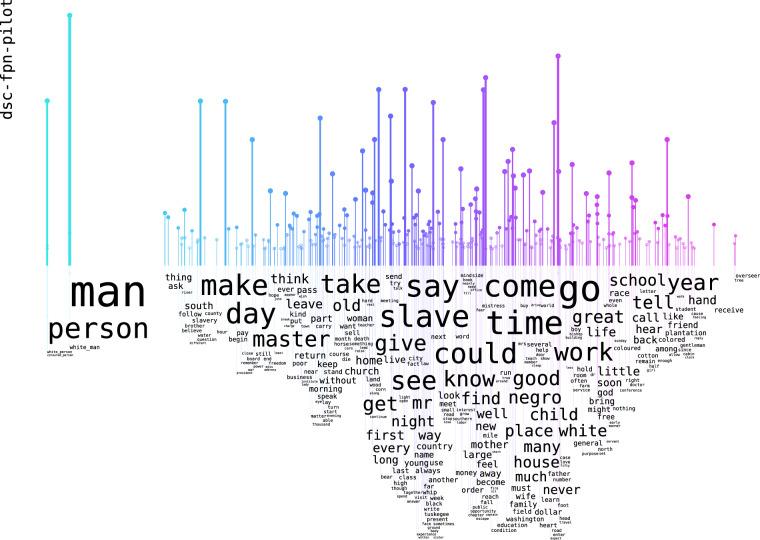
Wordrain of Word2vec analysis from the DSC first person narrative sub-corpus: both “man” and “person” can be seen used for different people, they are in the same vertical space as both “white_man” and “colored_person”.

**Fig. 5:**
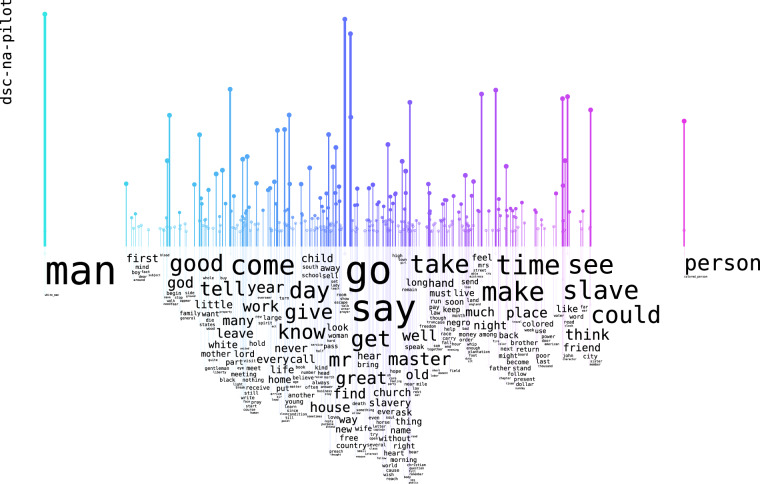
Wordrain of Word2vec analysis from DSC other narratives sub-corpus: “man” and “person” are used differently for different people, they are on the opposite sides, with “white_man” in the same vertical space as “man” and “colored_person” in the same space as “person”.

The content can be explored through word rain^12^ analyses, as Figs. 4, 5 show. These semantic vector analyses were conducted on the DocSouth texts, to get an idea of the content. The models for these are available at our repository (10.23695/P5HW-DR52). In word rains, words that have a similar semantic meaning are located in close proximity to each other. A preliminary finding from the vector analysis is the presence of a discernible semantic distinction between the nouns “man” and “person” in two of the different subsets, both originating from Documenting the American South.

As illustrated in Fig. 4, in the first person narratives of formerly enslaved persons, the nouns “man” and “person” can be used to refer to any person, irrespective of their origin. As illustrated in Fig. 5, which is based on other narratives from individuals of diverse backgrounds, the noun “man” most often describes white persons, whereas “person” most often describes people of African-American descent.

## Usage Notes

Corpus version 0.1^5^ is based on the data in our pilot project, and later versions will contain more data and more analyses. On the referenced page, there are more detailed usage notes, and included in the dataset are “Read me” files. There is also a specific folder for instructional files and films in the repository folder “Documentation”.

## Data Availability

The data set and other resources are available at Språkbanken Text’s resource site (10.23695/P5HW-DR52).

## Code availability

The code and data for the Volces of formerly Enslaved Corpus is publicly available under a Creative Commons Attribution-ShareAlike 4.0 International license (CC BY SA 4.0). This is to make it as available as possible to others, since the data are part of the public domain and human cultural heritage.
